# Quantifying Impairments in the Subacute Phase of Whiplash Associated Disorders—A Cross-Sectional Study

**DOI:** 10.3390/life15040562

**Published:** 2025-03-31

**Authors:** Harpa Ragnarsdóttir, Guðný Lilja Oddsdóttir, Magnús Kjartan Gíslason, Kristín Briem

**Affiliations:** 1 Research Centre of Movement Science, Department of Physiotherapy, University of Iceland, 102 Reykjavík, Iceland; glo@hi.is (G.L.O.); kbriem@hi.is (K.B.); 2 Elja Physiotherapy, 220 Hafnarfjordur, Iceland; 3 Institute of Biomedical and Neural Engineering, Reykjavik University, 102 Reykjavik, Iceland

**Keywords:** whiplash, car accident, car collision, WhipPredict, movement control, position sense, cervical range of motion, proprioception

## Abstract

Whiplash-Associated Disorders (WADs) often result from traffic accidents, leading to persistent symptoms, including neck pain, disability, dizziness, and central sensitization (CS). A key concern is cervical range of motion (cROM) impairment and sensorimotor dysfunction, which contribute to prolonged disability. This study assessed functional performance in individuals with subacute (>1, <3 months) WADs (n = 122) compared to healthy controls (n = 45). Clinical measures included cROM, movement control (Butterfly test), and position sense (Head–Neck Relocation Test, HNRT). Patient-reported outcomes included neck disability, pain intensity, central sensitization, and dizziness. Mixed and linear models evaluated group differences and the influence of demographic and symptom-related factors. WAD patients had significantly reduced cROM and impaired movement control (*p* < 0.001). Neck disability (*p* < 0.001) and pain intensity (*p* = 0.015) affected cROM within the WAD group. Interaction effects revealed greater amplitude accuracy (AA) impairments at greater difficulty levels (*p* = 0.043), while time on target (TOT) differences decreased (*p* < 0.001). Dizziness was associated with increased undershoot (*p* < 0.001), while pain negatively impacted both AA (*p* = 0.003) and TOT (*p* = 0.037). Position sense did not differentiate WAD patients from controls. Findings suggest task-dependent sensorimotor deficits, highlighting the need for multimodal assessment. Early CS screening may optimize rehabilitation and prevent chronic disability.

## 1. Introduction

The primary cause of a whiplash injury is energy transfer to an individual’s neck via an acceleration–deceleration inertial force such as one seen in traffic accidents (TAs) [1]. The injury can cause damage to bony structures and soft tissues at the cervical spine level resulting in a variety of chronic musculoskeletal-, psychological-, and neurological symptoms that are collectively named Whiplash-Associated Disorders (WADs) [1]. WADs following a TA have been a matter of interest and research for years as these disorders tend to become persistent [2,3]. When treating individuals with WADs, it is imperative that all symptoms be taken into account early on in the rehabilitation process, promoting a biopsychosocial approach [4].

Cervical range of motion (cROM) has been found to be decreased in individuals with WADs [5], both compared to healthy controls, and to individuals with chronic non-traumatic neck pain [6,7]. In addition, sensorimotor control disturbances following TAs have been reported in patients with WADs, as reflected by dizziness, impaired joint position sense, and poor balance in standing [8]. Research has shown that sensorimotor integration is likely affected due to impaired cervical proprioception, as opposed to impairments in the central nervous system (CNS) or vestibular system [9]. However, while cervical proprioceptive deficits are considered a key contributor to impaired sensorimotor integration in WADs, involvement of the vestibular system and its central processing pathways has also been suggested [10]. Additionally, whiplash injuries may co-occur with mild traumatic brain injury, which could further influence sensorimotor function [10]. The function of the deep neck muscles, rich in proprioceptors, is known to be impaired in WAD patients [11,12]. In addition to being crucial for cervical mobility and stability, because of their substantial amount of proprioceptors, they play a vital role in the functioning of the sensorimotor control system, as well as the vestibular and visual systems [9,13]. Proprioception refers to the body’s ability to perceive its own position in space, and includes the senses of position, movement, and balance [14,15]. Females typically exhibit less neck strength compared to males and significantly smaller external neck and vertebral dimensions [16], which may contribute to increased susceptibility to whiplash injuries and possibly greater vulnerability toward impairment in the proprioceptive system.

Reduced cROM [5,6,7,17] and impaired proprioception [8,18,19,20] are well documented in individuals with acute and chronic WADs. Moreover, greater neck pain and disability have been negatively associated with both cROM [17] and proprioception [21]. Additionally, central sensitization (CS) has commonly been associated with chronic [22,23,24] WADs as it follows an ongoing nociceptive input and neuroplastic changes in the CNS [25]. A heightened sensitivity to pressure pain [26] and cold pain [26,27,28,29,30] in individuals with acute WADs compared to healthy controls has been reported and linked with chronic disability, suggesting the involvement of CS. Furthermore, research in musculoskeletal pain populations suggests that symptoms of CS, as measured by the Central Sensitization Inventory (CSI), can serve as predictors of pain-related disability over time, with more severe CS symptoms associated with greater disability at follow-up [31]. When it comes to the subacute phase of WADs, research on dizziness and CS and their effect on proprioceptive ability is lacking. The subacute phase has been described as a subset of the acute phase, involving pain that is longer-lasting than the acute pain but not yet chronic [32]. Central sensitization and dizziness are known to be present in a big proportion of chronic WAD patients and those symptoms may lead to changes in the CNS or the somatosensory system with secondary, negative effects on proprioceptive performance [22,23,24,33]. The subacute phase could provide a critical window of opportunity to prevent long-term complications as the focus shifts from reducing inflammation and pain and preventing further injury to promoting tissue healing, restoring function, and reducing disability.

Quantifying impairments of the head and neck and understanding their association with patient-reported outcomes is essential for assessing the severity of WADs and providing individualized and multimodal treatment strategies. Adding sensorimotor training to individual treatment programs (in addition to neck-specific exercises and manual therapy) can maintain improvements in neck pain and disability in the long term [34]. In recent years, virtual reality [35,36,37,38,39,40,41] has increasingly been used to test sensorimotor control [42,43,44]. The aims of this study were to (1) compare cROM, movement control, and position sense between individuals with subacute WADs and healthy controls and evaluate the effect of sex and age; and (2) assess the effect of CS, neck pain and disability, dizziness, sex, and age on functional measures. Individuals with WADs were expected to show worse functional performance compared to healthy controls, older individuals were expected to show decreased ROM in addition to worse movement control and position sense compared to younger, and females were expected to show worse movement control and position sense than males irrespective of group. Neck disability and pain were expected to be negatively associated with all functional measures, while dizziness and CS were expected to be negatively associated with both movement control and position sense.

## 2. Materials and Methods

A cross-sectional study consisting of individuals with subacute (>1 month, <3 months) WADs following a car collision (WAD group) and healthy individuals (control group) was conducted in a private PT clinic in Kopavogur, Iceland. Inclusion criteria for the WAD group were WAD grades I–II [1] verified by WhipPredict [45,46], with medium-to-high-risk symptoms, age ≥ 18 years old. Excluded were individuals showing a considerable degree of known or suspected physical pathology (i.e., myelopathy, spinal tumors/infections/surgery, ongoing malignancy), those who had severe neck problems resulting in sick leave for >1 month in the year prior to the current injury and those who lacked the ability to read/write Icelandic. Inclusion criteria for the control group were neck disability < 14% measured with the Neck Disability Index (NDI) and age ≥ 18 years old. Participants in the WAD group were recruited through the databases of the Emergency Department of Landspitali University Hospital (LUH), Reykjavik, Iceland, and the Health Care Centers (HCC) of the Capital Area over a one-year period (May 2022–May 2023). The trial was part of a bigger treatment study that was approved by the National Bioethics Committee (Iceland) and the protocol was registered (NCT05319808) and published prior to the end of the recruitment [47].

All participants signed an informed consent form, and all outcome measures were collected by the same clinician/researcher. All participants answered the NDI [48], a 10-item self-report questionnaire, scored on a 0–5 Likert scale, with strong reliability and validity [49,50,51] for assessing neck pain-related disability. In addition, the WAD group was assessed with a 100 mm Visual Analog Scale (VAS) for average neck pain intensity over the past week (0 = no pain, 100 = worst imaginable pain), a measure widely used and validated in musculoskeletal populations [49,52]. Dizziness-related disability was evaluated using the Dizziness Handicap Inventory (DHI) [53] a 25-item scale with established reliability and validity [53,54], scored 0–100. Central sensitization symptoms were assessed with the Central Sensitization Inventory (CSI), a 25-item tool using a five-point Likert scale (0–4). The CSI has shown good psychometric properties in chronic pain populations [55,56]. For all questionnaires, a greater score represented greater disability or symptom burden.

**Figure 1:**
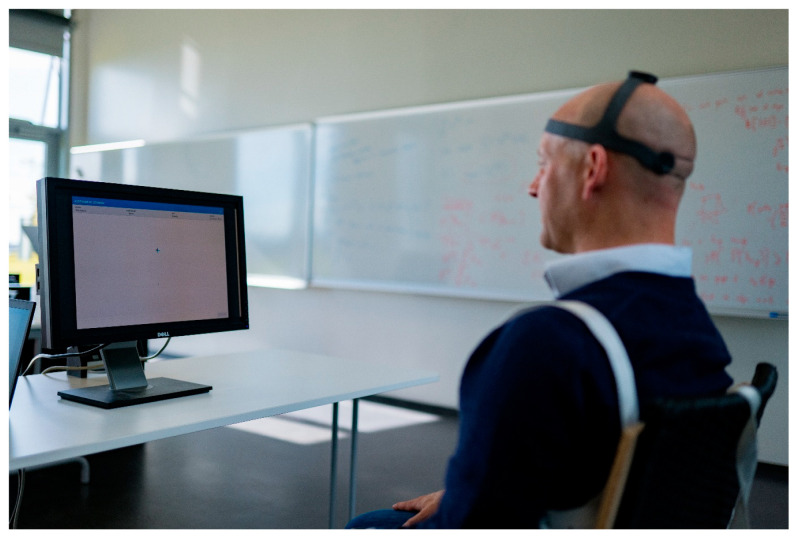
Experimental setup (the photo is published with consent from the man on the photo).

**Figure 2:**
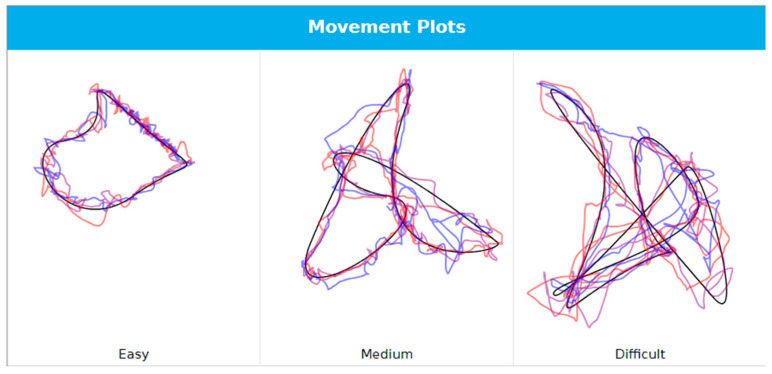
Three unpredictable trajectories (black line) used in the Butterfly test for cervical movement control and an example of a participant’s performance (colored lines).

The functional measures were collected using the NeckCare system. A neck gear/plastic helmet, with a 3D sensor that tracks the cervical and head position/movement in space was used to assess cROM, movement control, and position sense (Figure 1). Participants were seated 90 cm from a computer screen with both feet on the floor and the shoulder girdle fixed by two straps over each shoulder. Head movements were measured during the execution of three (movement) tests, the order of which was randomized using the Fisher–Yates shuffle algorithm. Each test was repeated three times:

1. The Butterfly Test (previously known as the Fly test) for cervical movement control is a reliable and valid test that has been proven to be fast and easy to use in a clinical setting [57] that can discriminate between healthy individuals and those with neck pain [19,40,58]. It consists of three different, unpredictable trajectories with increasing difficulty (easy, medium, and difficult, Figure 2) determined by the geometry of the movement tasks, the velocity of the target, and the length of the trajectories, as described by Oddsdottir et al. [58]; however, the trajectories used in this study were approximately three times smaller than in previous research, with range of motion 10° in each direction (diameter: 20°). Participants were instructed to track the trajectories by following a small red circle as accurately as possible, aiming at keeping the cursor within the red circle, and by using head and neck movement to manipulate an on-screen cursor. Metrics included (1) the Amplitude Accuracy (AA): the absolute average distance (radius) in arbitrary length units between the cursor that represents the head position and the target, where a lesser value represents a better score; and (2) the percentage of time the cursor spends: (a) in a mathematically determined, invisible free zone around the target (Time on Target, TOT); (b) ahead of the target (Overshoot, OS); or (c) behind the target (Undershoot, US). Each trajectory was repeated three times.
2. The Whole Cervical Range of Motion Test for maximum active ROM in all 3 planes measured in degrees. Each movement (flexion–extension (sagittal), left/right rotations (transverse), and left/right lateral flexions (frontal)) was repeated three times, and the maximum value for each direction in each plane was used for analysis for maximum total movement in each plane.
3. The Head Neck Relocation Test (HNRT), which assesses head–neck position sense, is derived from the cervical Joint Position Error Test (cJPT) [59]. The cJPT is a validated tool capable of distinguishing between healthy individuals and those with neck pain [60,61] as well as between healthy individuals and those with acute or subacute WADs [62]. The test was conducted both in the sagittal plane (flexion/extension) and the transverse plane (left/right rotation). Participants were blindfolded to eliminate visual input and asked to locate their perceived neutral head position. Once identified, they were instructed to move their head as far as comfortably possible in the specified direction and then return to what they believed was the original neutral position. Participants verbally indicated when they felt like they had returned to neutral by saying ‘ok’, at which point the examiner recorded the position with a spacebar click. If necessary, the examiner repositioned the participant to the original neutral position between the trials. The outcome measure was the absolute error, defined as the angular difference in degrees between the actual and perceived neutral positions.

Data were analyzed in Jamovi statistical software, version 2.3.28, independent of sponsors and competing interests. The WAD group was recruited as part of a larger clinical trial cohort, while the healthy control group was recruited separately. For the Butterfly metrics, the means for all three repetitions of each difficulty level were used for data analysis.

In addition to descriptive statistics, mixed model ANOVAs were used to assess performance in movement control using Butterfly test difficulty levels (easy, medium, difficult), gender (male vs. female), and groups (WAD vs. control) as factors. Similarly, general linear models were used to assess active cROM and position sense (HNRT) using groups (WAD vs. control) and gender (male vs. female) as factors. For position sense (HNRT), the corresponding available active cROM was used as a covariate for each movement. In all the above models, age was used as a covariate to assess if it would affect the models. For within-group differences, scores on questionnaires (NDI, VAS, DHI, CSI) were also used as covariates to assess if they would affect the models, and when there was no effect (did not change statistically significant results), they were removed, and results reported. Significant interactions were represented using *p* values (<0.05) and F values (represented along with their degrees of freedom). Bonferroni was used for post hoc comparisons. When data were not normally distributed (using the Kolmogorov–Smirnov test and residual histogram assessment), they were transformed to log(10) before mixed-model or general linear model analysis. For all objective measures, extreme outliers were removed as errors when exceeding physiological limits of movement.

## 3. Results

**Figure 3:**
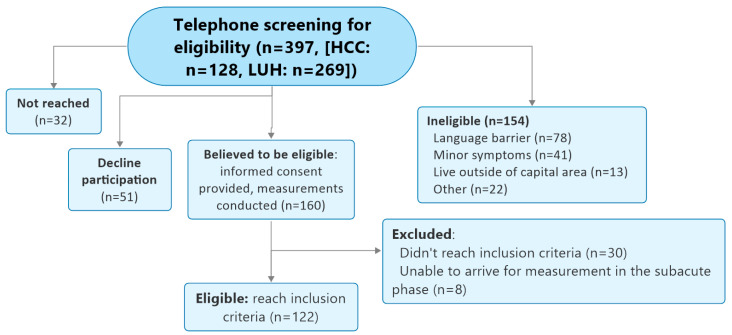
Flow diagram of the trial protocol. Abbreviations: HCC: Health Care Centers of the Capital Region, LUH: Landspitali University Hospital.

A total of 122 individuals (mean age 40.5 years, SD 13.1, range 18–69, IQR 29.5–49, 90th percentile 58.9 years; 59% female) consented to participate and met criteria for inclusion in the WAD group after a mean of 46.1 (SD 15.9, range 28–93) days since the TA (Figure 3). Most of the individuals were drivers in their car (81%) and most had been in a rear-end collision (45%) as opposed to a rear- and front- (8%), front- (20%), or side- (25%) collision or an overturn (3%). Approximately half of the individuals did not see the collision coming (54%) and 38% were stationary when the collision happened. Most (n = 92, 75%) had not submitted a claim to their insurance companies. The control group consisted of 45 age-matched healthy individuals (mean age 37.3 years, SD 11.6, range 23–66, IQR 29–41, 90th percentile 57.4; 55.6% female). Table 1 and Table 2 show mean scores for both groups in self-reported and objective measurements.

### 3.1. Between Group Differences

**Figure 4:**
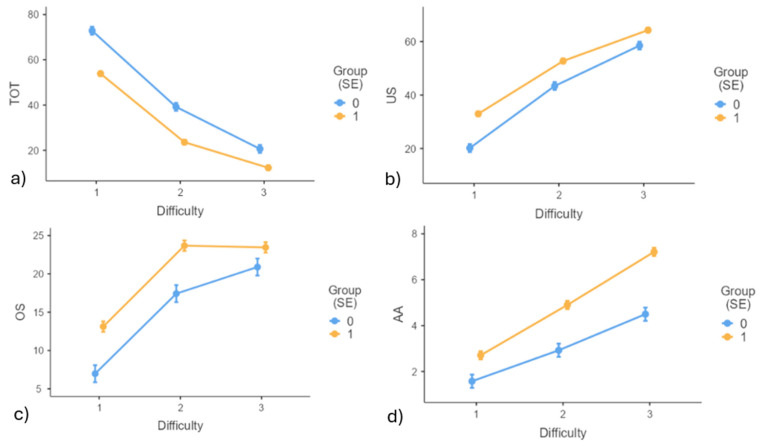
Mean performance measures, with statistically significant Group difficulty level interactions, on the Butterfly test for (**a**) Time on Target (TOT), (**b**) Undershoot (US), (**c**) Overshoot (OS), and (**d**) Amplitude Accuracy (AA). 0 = controls, 1 = WAD group. Difficulty levels include (1) easy, (2) medium, and (3) difficult.

An expected main effect of difficulty level was found for all metrics of the Butterfly test (better performance for easier levels; *p* < 0.001), as well as a main effect of group for all metrics (better performance of controls; *p* < 0.001). In addition, a significant interaction was found for Butterfly test performance (group*difficulty level) as the magnitude of group differences depended on the difficulty level (Figure 4). On average, differences in TOT (F(2326) = 15.181, *p* < 0.001), US (F(2326) = 37.45, *p* < 0.001) and OS (F(2326) = 8.70, *p* < 0.001) between healthy and WAD participants decreased with greater difficulty levels while the opposite held true for AA (F(2325) = 3.167, *p* = 0.043) where the drop in accuracy was more dramatic for WAD participants compared to controls. Group differences were not influenced by gender or age for any Butterfly metrics.

For cROM, an expected main effect of group was found for all planes (less ROM of WAD patients; *p* < 0.001) where about a 15° difference was seen in the sagittal and transverse planes, but less in the frontal plane (Table 2). A main effect of age was evident (reduced ROM with higher age) in the frontal (*p* < 0.001, ⴄ^2^p = 0.187) and sagittal (*p* = 0.039, ⴄ^2^p = 0.028) planes but not in the transverse plane. Gender had no influence on cROM.

Interestingly, when assessing position sense, the only between-groups difference was found during HNRT performance for left rotation (*p* = 0.027, ⴄ^2^p = 0.032) and extension (*p* = 0.046, ⴄ^2^p = 0.027) where controls showed slightly less error (Table 1). Neither gender nor age influenced position sense.

### 3.2. Specific Analyses of WAD Participants

When Butterfly data of WAD participants were analyzed, an expected main effect was seen for difficulty levels for all metrics of the test due to better performance for easier levels (*p* < 0.001). Interestingly, pain levels influenced both AA (F(1326) = 7.89, *p* = 0.003) and TOT (F(1114) = 4.32, *p* = 0.037) revealing a worse performance with a greater VAS pain score regardless of difficulty level. Performance was also influenced by the DHI score (greater US with a greater DHI score; F(1277) = 14.405, *p* < 0.001), and age (greater OS with younger age F(1113) = 7.82, *p* = 0.005). Gender had no influence on Butterfly performance and Butterfly metrics were not affected by neck disability or CS.

For active cROM, sagittal and transverse mobility was reduced with a greater NDI score (*p* < 0.001, ⴄ^2^p = 0.263 and ⴄ^2^p = 0.292, respectively), whereas frontal plane motion was not affected. Greater pain (VAS) was associated with a mild reduction in frontal plane mobility alone (*p* = 0.015, ⴄ^2^p = 0.053). Overall, male WAD participants had slightly less total rotation excursion than females (main effect; *p* = 0.025, ⴄ^2^p = 0.044), while no difference was seen in other planes of motion. Overall, age negatively influenced total cROM in the transverse (*p* = 0.006, ⴄ^2^p = 0.068) and frontal (*p* < 0.001, ⴄ^2^p = 0.222) planes but not the sagittal plane. Dizziness and CS did not affect any ROM. When assessing the HNRT metrics, no difference between sexes was found and no influence was noted from any of the questionnaire scores, from age, or from the corresponding active cROMs.

## 4. Discussion

Research on sensorimotor and cROM impairments in the subacute phase of WADs is limited, despite its relevance for early rehabilitation and prevention of chronic disability. This study aimed to address this gap by comparing cROM, position sense, and movement control between individuals with subacute WADs and healthy controls and by assessing the influence of CS, neck pain, disability, dizziness, sex, and age on functional performance. The study’s results generally confirm that sustaining neck injuries in a TA results in significantly impaired cROM and movement control and increased disability, as reflected by performance on functional measurements and NDI scores. The effects of age and gender on functional measurements were minimal, as was the influence of patient-reported measures.

The interaction between groups and difficulty levels on the Butterfly test revealed progressively worse performance with increasing difficulty. The magnitude of group differences varied depending on the metric. While differences in movement control performance between healthy controls and WAD patients lessened for TOT at greater difficulty levels, they increased significantly for AA. This aligns with previous research [20,40] suggesting that dysfunction in WAD patients is more evident in tasks requiring precise adjustments, such as more complex trajectories. The smaller group differences in TOT at greater difficulty levels likely reflect the general challenge of difficult trajectories for all participants. In contrast, larger group differences for AA may stem from WAD patients deviating more during turns. This can create brief but substantial deviations that increase AA without greatly affecting TOT. These results have important rehabilitation implications. While improving TOT is important, AA should also be a key focus of intervention, particularly under greater difficulty levels. Impaired proprioceptive signals or oculomotor dysfunction may contribute to poorer AA by disrupting the cervicocollic [63], or cervicoocular reflexes [64] and, therefore, the ability to accurately follow the target. Understanding and addressing these dysfunctions could enhance training strategies for improving both AA and TOT. However, it is worth noting that AA and TOT are inherently linked; large TOT typically leads to reduced AA, and vice versa. Training them separately may be challenging. Instead, targeting underlying mechanisms, such as proprioceptive and oculomotor function, may enhance performance across both metrics.

The results from this study suggest that the HNRT may not effectively differentiate between healthy individuals and those with subacute WADs. This contrasts prior research, where a meta-analysis [8] found significant differences in joint position error between WAD patients and controls. The lack of significance in this study may stem from differences in measurement methods, researcher accuracy, or time since injury. Previous studies largely focused on chronic WADs, whereas this study examined the subacute phase. Although the groups in this study were not matched 1:1, previous studies investigating proprioceptive impairments in similar populations have shown that approximately 30 individuals per group are sufficient to detect meaningful group differences [19,65,66,67,68,69,70]. Additionally, a post hoc sample size calculation using G*Power, version 3.1.9.7, indicated that a total sample of 128 (64 per group) for the general linear models and a total sample size of 28 (14 per group) for the mixed models would be sufficient to detect medium effect sizes and 80% power. However, the actual group sizes in this study (WAD = 122, control = 45) provided approximately 65% power for the general linear model comparisons, indicating reduced sensitivity for detecting between-group differences.

Central sensitization did not significantly affect movement control, position sense, or cROM. Within WAD participants, dizziness was associated with increased US in the Butterfly test, possibly due to conservative movement strategies, reduced movement speed, or underlying mechanical dysfunction, such as cervical facet or uncovertebral joint dysfunction. Overshoot, typically indicative of poor deceleration or overcompensation, was not directly affected by dizziness. Pain negatively impacted both AA and TOT, suggesting that it interferes with the ability to maintain steady movements (TOT) and increases deviations from the target (AA). This supports prior research indicating that pain can alter proprioception and lead to compensatory movement strategies, which may persist even after the pain has resolved [71]. Neck disability only affected cROM in transverse and sagittal planes, not the frontal plane, while the opposite held true for pain levels. Prior research supports this association [72].

The results showed limited effects of gender and age on functional performance. Consistent with previous studies [73,74], no significant gender differences were found for cROM, except for a slight reduction in rotation ROM in males within the WAD group. This may be due to anatomical factors, such as lower muscle mass or smaller external neck and vertebral dimensions in females compared to males [16], though the difference was small. The expected decline in ROM with age [73,75,76,77] was observed, but uneven age distribution may explain the lack of significant age effects in some planes of motion.

The results indicated that even in the subacute phase, WAD patients may have developed severe CS, mild to moderate dizziness, and moderate neck disability. Identifying the presence of dizziness at this early stage is important as it is more often a symptom reported in late stages [8]. Similarly, CS has mostly been associated with chronic WADs [22,23,24] as opposed to acute [26,78] or subacute WADs where the literature seems to be limited. Its presence in the subacute phase indicates the need for early screening. Persistent nociceptive input following TAs may trigger CS, amplifying pain responses and increasing the risk of chronic pain conditions [79]. Screening for CS in the subacute phase could serve as a predictor of chronicity and guide targeted interventions to reduce the risk of long-term symptoms. However, further research is needed to clarify the role of CS in the subacute phase of WADs and to inform the development of effective interventions.

The limited impact of patient-reported outcomes (e.g., neck disability, pain, dizziness, and CS) on functional performance measures suggests that self-perceived symptoms may not fully capture the extent of functional impairment. This aligns with previous research that indicated limited correlations between self-reported and objective measures [17,80,81]. Questionnaires may be influenced by factors such as pain tolerance, psychological state, and personal experiences, whereas objective measures provide standardized results that are less biased [82]. Therefore, a combination of patient-reported and functional assessments is essential to ensure comprehensive and personalized care for WAD patients.

### Limitations

This study has several limitations. First, although the sample size was adequate based on prior studies [19,65,66,67,68,69,70], a relatively high number of eligible individuals declined to participate or were unreachable, raising the possibility of selection bias, and potentially limiting the generalizability of the findings. Second, while our total sample size was sufficient for most comparisons, the group sizes between WAD and control participants were unbalanced, and a post hoc power analysis revealed that we did not achieve adequate statistical power for comparisons between WADs and control on the HNRT and cROM outcomes. The power for these comparisons was only 65%, indicating an increased risk of type II error. Future studies should aim for more balanced group sizes to strengthen the validity of between-group comparisons. Third, participants were not systematically screened for structural brain injury, vestibular impairment, post-traumatic myelopathy, or muscular and mechanical lesions of the neck, all of which may influence sensorimotor and functional outcomes in whiplash-associated disorders (WADs) [9,10,11,69,83]. Fourth, although central sensitization (CS) was evaluated using the Central Sensitization Inventory (CSI), no additional psychometric measures such as the Tampa Scale for Kinesiophobia or anxiety questionnaires were included, which may have provided further insight into psychological and behavioral contributors to function. Moreover, the CSI may be less specific in the subacute phase, and elevated scores should be interpreted with caution. Fifth, only gross cervical range of motion (ROM) was assessed; specific joint-level limitations, which may be relevant in persistent neck pain [84], were not evaluated. Future research should consider including more detailed neurological, vestibular, and musculoskeletal assessments; additional psychological screening tools; and strategies to minimize participation bias to improve generalizability.

## 5. Conclusions

This study provides important insights into functional performance in individuals with subacute whiplash-associated disorders (WADs). Participants with WADs showed a reduced cervical range of motion (cROM) and poorer movement control compared to healthy controls, especially as task difficulty increased. Importantly, symptoms of central sensitization (CS), dizziness, and neck-related disability were already present at this early stage, underlining the value of early screening for risk stratification and targeted intervention. However, there were no significant group differences in position sense (HNRT), in contrast to previous research. The limited associations between patient-reported outcomes (e.g., pain, disability, CS, and dizziness) and objective measures such as cROM and movement control support the use of both self-reported and functional assessments in clinical decision-making. Together, these findings reinforce the complexity of WADs in their subacute phase and highlight the importance of comprehensive, multimodal evaluation and early management to potentially prevent the transition to chronicity.

## Figures and Tables

**Table 1:** Means and standard deviations (SD) for AA (arbitrary length unit) and TOT, OS, and US (%) for movement control (Butterfly test), and position sense (HNRT, degrees).

			Mean (SD)	
			WAD Group (n = 122)	Healthy Controls (n = 45)
Butterfly	Easy	AA	2.8 (1.3)	1.6 (0.3) *
		TOT	53.6 (17.0)	72.9 (6.0) *
		OS	13.4 (13.2)	7.0 (2.6) *
		US	33.1 (7.5)	20.1 (5.0) *
	Medium	AA	5.0 (2.2)	2.9 (0.5) *
		TOT	23.3(12.5)	39.2 (9.8) *
		OS	23.9 (9.2)	17.5 (5.7) *
		US	52.8 (10.1)	43.3 (7.6) *
	Difficult	AA	7.3 (2.9)	4.5 (0.8) *
		TOT	12.0 (7.0)	20.6 (6.5) *
		OS	23.7 (7.5)	20.9 (6.7) *
		US	64.3 (7.8)	58.4 (7.7) *
Head Neck Relocation	Flexion		2.8 (1.9)	2.7 (1.8)
	Extension		3.1 (2.0)	2.9 (1.9) *
	Left rot		2.8 (2.0)	2.0 (1.3) *
	Right rot		3.1 (1.9)	3.1 (1.6)

* Statistically significant (*p* < 0.05) Abbreviations: AA: Amplitude Accuracy, TOT: Time on Target, OS: Overshoot, US: Undershoot.

**Table 2:** Means and standard deviations (SD) for range of motion (degrees) and self-reported measurements (scores ranging from 0 to 100).

	Mean (SD)	
	WAD Group (n = 122)	Healthy Controls (n = 45)
Flexion	45.2 (13.7)	60.1 (8.9) *
Extension	50.9 (16.8)	65.5 (11.9) *
Left rot	58.0 (14.2)	73.4 (8.4) *
Right rot	57.3 (14.7)	74.0 (7.3) *
Left lat flex	31.7 (8.8)	39.2 (8.2) *
Right lat flex	31.7 (9.3)	32.8 (8.2) *
NDI	42.3 (19.3)	3.4 (3.7)
VAS	6.8 (2.1)	NA
CSI	49.7 (16.8)	NA
DHI	35.2 (25.3)	NA

* Statistically significant (*p* < 0.05); NA: Not applicable for the healthy group; Abbreviations: lat flex: lateral flexion, NDI: Neck Disability Index, VAS: Visual Analog Scale, CSI: Central Sensitization Inventory, DHI: Dizziness Handicap Inventory.

## Data Availability

Data can be accessed upon reasonable request from the lead author.

## Abbreviations

The following abbreviations are used in this manuscript:

WAD	Whiplash-Associated Disorder
TA	Traffic accident
cROM	Cervical range of motion
CNS	Central nervous system
CS	Central sensitization
NDI	Neck Disability Index
LUH	Landspitali University Hospital
HCC	Health Care Center
VAS	Visual Analog Scale
DHI	Dizziness Handicap Inventory
CSI	Central Sensitization Inventory
HNRT	Head–Neck Relocation Test
JPE	Joint position error
cJPT	Cervical Joint Position Test
ANOVA	Analysis of variance
OS	Overshoot
US	Undershoot
TOT	Time on target
AA	Amplitude accuracy
